# Developing new possibilities for interprofessional learning- students’ experience of learning together in the ambulance service

**DOI:** 10.1186/s12909-022-03251-8

**Published:** 2022-03-20

**Authors:** Helen Conte, Jonas Wihlborg, Veronica Lindström

**Affiliations:** 1 Department of Neurobiology, Care Sciences and Society, Division of Nursing Karolinska Institutet, Stockholm, Sweden; 2 School of Health and Welfare, Dalarna University, Falun, Sweden; 3 Department of Health Promotion, Sophiahemmet University, Stockholm, Sweden; 4 Samariten Ambulance Stockholm, Stockholm, Sweden

**Keywords:** Interprofessional learning, Emergency medical services, Mixed method

## Abstract

**Background:**

It is known that setting and context matters, and contextual factors influence interprofessional education (IPE). Activities developed in a new setting should therefore be evaluated to determine students’ experiences and learning. IPE in the ambulance service may present a new setting for interprofessional learning (IPL).

**Aim:**

The aim of this study was to explore undergraduate students’ experiences of collaboration and learning together during their clinical rotation in the ambulance service.

**Study design and method:**

A mixed convergent parallel design was used to describe nursing and medical students’ experiences of collaboration and learning together during their clinical rotation in the ambulance service during autumn 2019. Two group interviews with nursing students (*n* = 20; response rate 80%) were conducted and the medical students (*n* = 40; response rate 72.5%) answered a self-assessment questionnaire regarding their IPE. The group discussions were analysed using an inductive thematic analysis and descriptive statistics were used to describe the medical students’ self-assessed experiences and competencies in interprofessional collaboration.

**Results:**

In the context of the ambulance service, some of the challenges included, the team vary daily, a context that can be unpredictable, and the team being required to make decisions in various situations with limited support. The context presented good opportunities to learn together, since they faced a broad variety of situations and had opportunities to follow patients through the chain of care.

**Conclusion:**

The students’ experiences show that the ambulance service offers possibilities for IPL. The ambulance service enhanced the students’ learning in an unfamiliar environment, encouraging them to develop collaborative learning strategies and situational leadership regardless of established hierarchical structures and stereotypes that are sometimes present in other parts of the health care service.

**Ethical approval:**

By the Swedish Ethical Review Authority. No: 2019–03595.

## Background

Interprofessional Education (IPE) should be integrated in health education as part of making future professionals ready to collaborate and to improve patient safety [1]. Research suggests that IPE occurs in diverse settings worldwide on both pre-graduate and continuous level [2]. There are challenges finding common ground for IPE activities between educational programmes; the reality challenges the vision, since the majority of health care education is provided in places and spaces that do not support either shared interplay or shared learning [3].

IPE includes all types of educational training where activities focus on more than one profession and where interprofessional learning (IPL) stems from joint and interactive group processes [4]. The students are active participants, and they learn with and from each other in authentic activities [5]. The groups often consist of nursing and medicine students overlapping with physiotherapy and occupational therapy students [2, 6, 7]. IPE can consist of a wide range of activities depending on the aims, educational setting, and availability of participants. Systematic reviews have identified a wide range of IPL activities, e.g., rotations at IPTWs, clinical scenarios, group discussions, simulator training, and community projects [8, 9]. Students in the community setting suggested that co-location alone is not enough for different professionals to learn together effectively, and facilitation is vital [10]. The supervisors are the key to setting realistic goals, motivating students, managing interactions, minimising friction, and ensuring equal participation in IPE [9]. The supervisors’ facilitative actions are supposed to be strategic and create opportunities for the learners to think, act, and reflect together as a team [11].

### Context

Researchers have systematically mapped IPE [2, 6–8], and have shown that setting and context matters [8] and contextual factors influence IPE [9]. Therefore, in each activity developed and implemented in different contexts the students’ experiences should be considered. Little is known about IPE in the ambulance service and it may present a new setting for IPL as it is recognised as presenting opportunities for undergraduate nursing students to learn about and with patients, and students can follow patients through the chain of care [12, 13]. In the chain of care, the ambulance team’s first encounter with the patient is through the dispatch of the ambulance assignment. The emergency medical communications centre (EMCC) conveys information to the ambulance team, including priority of assignment and an assessment of the patient’s illness/injury. The next step in the chain of care is when the ambulance team enter the scene [14], assess medical and nursing needs, initiate symptom alleviation and/or medical treatment, and decide on further care needed and optimal level of care for the patient [15]. After decisions are made, the patient is transported to the next level of care [16] or stays at the scene with self-care advice [17]. During transport to the next level of care, continuous assessments, medical and nursing interventions are carried out as needed. The ambulance team’s participation in the chain of care usually ends with the handover of the patient to the next level of care, e.g., emergency department (ED) or other health care facility [16]. It has been suggested that the ambulance service may be suitable for IPE, since patients present with a variety of symptoms and with both nursing and medical needs [18, 19]. In addition, supervisors are available to facilitate IPE for the duration of the ambulance assignment. An ambulance team consists of one registered nurse (RN) with 1 year of additional training in emergency care and one emergency medical technician (EMT) with basic life support competence [16]. In addition, there are physician-manned units in the emergency medical services (EMS) and medical supervisors at the EMCC assisting the ambulance team as needed. Examples of assignments where the ambulance team and physicians in the EMS work together include cases of cardiac arrest, child illness, birth, and other severe and/or life-threatening situations. The medical supervisors at the EMCC support the ambulance team in decision making concerning, e.g., non-conveyance patients. Altogether, this setting provides the required conditions for IPE for nursing and medical students. Therefore, the aim of this study was to explore undergraduate students’ experiences of collaboration and learning together during their clinical rotation in the ambulance service.

## Methods

A mixed convergent parallel design [20] was used to describe nursing and medical students’ experiences of collaboration and learning together during their clinical rotation in the ambulance service during autumn 2019.

### Setting

The study was conducted in Stockholm, Sweden. The county council is responsible for the EMS, and the ambulance service is provided by the organisation within the county and by private companies contracted by the county council. All ambulance assignments are dispatched by the EMCC answering the national emergency number 112. Nursing students from one university completed 6 weeks of clinical rotation in the ambulance service during their final semester in the study programme to become a RN. Medical students (semester 5, 6, 7 and 10) from another university were offered 2–4 days of clinical rotation with IPE in the ambulance service. Clinical supervisors (nurses and EMTs) were offered one educational day concerning IPE during 2018, which covered an introduction to IPE (2 h), clinical experiences of IPE supervision from two different contexts (IPTW and intensive care unit) (2 h), and follow-up discussions on how to handle potential aggravating situations that could occur in the ambulance service (2 h), e.g., traffic accidents. In total, eight clinical supervisors of 60 eligible (13%) participated in the educational day. No physicians participated since they are not employed by the ambulance service.

### Participants and data collection

Two group interviews with nursing students were conducted in January 2020 after they had finished their clinical rotation in the ambulance service and had received their grades (all passed). The group discussions started with a presentation of the study aim, information on recording the discussions, and confirmation that participation was voluntary. One of the group discussions was led by one moderator and one assistant, and the other group discussion was led by one moderator. The group discussions were based around four open-ended questions exploring the participants’ experiences of learning together in the ambulance service. The questions were: 1) *‘Can you tell me about your experiences of IPE during your clinical rotation in the ambulance service?’,* 2) *‘How did you work and learn together?’,* 3) *‘How did the supervisors facilitate the work and learning?’,* and 4) *‘What is your overall experience of doing clinical rotation and taking part in IPE in the ambulance service?’*. In addition, probing questions such as *‘Can you give examples?’, ‘Can you elaborate more?’,* and *‘Does anyone else have any other thoughts?’* were used to deepen and clarify the discussions. The discussions were audio recorded and lasted 40 and 43 min, respectively. The medical students were not able to participate in the group discussions due to scheduling issues; instead, they answered a self-assessment questionnaire in connection with their IPE. The self-assessment questionnaire consisted of three demographic questions (age, gender, previous experience of IPE), one open-ended question: *‘Overall, what is your experience of your and the team’s interprofessional collaboration during your clinical rotation in the ambulance service?*’, and questions regarding six domains of IPE; communication (4 items), collaboration (3 items), roles and responsibilities (5 items), collaborative patient/client-family centred approach (4 items), team functioning (5 items), and conflict management/resolution (3 items). The domains were inspired by and developed from the ICAR (Interprofessional Collaborator Assessment Rubric) [21]. A Likert scale was used to measure the students’ self-assessed competencies in interprofessional collaboration. The participants rated their agreement with a statement on a four-point Likert scale ranging from 1= “Never or completely disagree” to 4= “Always or agree completely”.

### Analysis

The transcribed group discussions were analysed using an inductive thematic analysis as described by Braun and Clarke where themes should be strongly linked to the data [22]. The analysis followed a six-step process where the last author (VL) led each step, and the first author (HC) reviewed the consistency and logic of the emergent themes. The whole process consisted of moving back and forth between the entire data set, the coded extracts, and the identified themes to ensure trustworthiness of the analysis. The first step consisted of listening to the recordings several times and repeatedly reading the transcribed discussions to ensure data immersion. The second step involved identifying and generating initial codes and text units and then coding and collating all data related to students’ experiences of IPE and learning together in the ambulance service. The coding was completed by the last author and checked back to the data set and discussed with the first author. The third step consisted of searching for patterns by gathering all codes into initial themes in an Excel spreadsheet. In the fourth step, the initial themes identified were corroborated through discussion and checking of the themes in relation to the codes using a thematic map. This fourth step was conducted in collaboration among all the authors to ensure rigour of the analysis. The fifth and sixth steps included, after discussions among all the authors, finalising the themes and presenting the findings.

Descriptive statistics and mean scores were used to describe the medical students’ self-assessed experiences and competencies in interprofessional collaboration. The interpretation of findings was then conducted in the discussion, in line with a convergent mixed method design [20].

## Results

### Nursing students’ experiences of IPE in the ambulance service

Sixteen out of 20 eligible (80%) nursing students (3 male and 13 female) participated in the group discussions. All had previous experience of IPE through simulation and seminars as part of their educational programmes. The two themes ‘*Learning by maintaining collaborative reasoning throughout the chain of care’* and *‘Factors facilitating learning and collaboration in the ambulance service’* underpin the IPL discussed by the undergraduate nursing students. Quotations from the group discussions is presented in Table 1.

**Table 1:** Quotations from the group discussions

Themes	Quotations
**Learning by maintaining collaborative reasoning throughout the chain of care**	*“We started talking already when we were given the assignment … what patient possible suffered of, and what to do when arriving ... at scene, the situation was not at all as we thought, we needed to re-think about how to collaborate, to be able to care for the patient ... we needed to use each other’s knowledge”* (Group 1) *“We reasoned together all the time, also during the care of the patient … we involved the patient in the discussion …*. *after the assignment we discussed with the supervisors about what was good and if we should have done something else”* (Group 2) *“It was so good ... just listening to the medical student and asking clarifying questions, I could understand how drugs and illness also affected the nursing and the patient’s developing of symptoms .... I could when caring for the patients also clarify different nursing aspects in the care for the medical student ...”* (Group 2) *“When the assignment did not turn out at all as we planned, it became the one who had the most knowledge ... skills ... who took the leadership, sometimes it was I and it felt a little strange that I should lead the medical student, but I did ... it was strange but nice …*” (Group 1) *“...during transport to the hospital we discussed and explained to the patient what has happening, what we have done and answered questions that patient asked ... in that way I could learn things that I had not thought about before”* (Group 1) *Sometimes it really did not work between me and the medical student, we did not talk to each other he/she was more interested in the phone than doing something together, I learned nothing ... I just wanted the shift to end …* (Group 2)
**Factors facilitating learning and collaboration in the ambulance service**	*“Meeting patients in the ambulance were totally different compared to previous clinical rotations … When I met patients at the ward there was always somebody else that had done the assessment and decided what I should do … in the ambulance I did it together with the medical student”* (Group 1) *“The supervisors were fantastic, they gave us the opportunity to learn by ourselves, they were in the background, I knew they were there ... it gave a me sense of security and at the same time a sense of working independently … after the assignment they* [supervisors] *challenged us with additional deepening questions and to reflect together”* (Group 2) *“Some of the supervisors were not good at all gave no support, questioned the IPL and students in the ambulance service in these situations I was happy to have a co-student … at least we had the possibility to learn together”* (Group 2) *“All the different assignments, not knowing what to expect meant that we* [students] *needed to discuss, otherwise it was difficult to work together and take care of the patient ... and when we needed help, the supervisors guided us”* (Group 1)

#### Learning by maintaining collaborative reasoning throughout the chain of care

The chain of care created IPL opportunities for the teams of students. The different activities along the chain – dispatch of assignment, pre-arrival to scene, at scene, and ending the care encounter – made it possible for the students to maintain joint clinical reasoning. The students focused on the patient’s ‘problems and plan of care’ and worked together to identify and reflect on their diverse knowledge and roles during the care encounter. The chain of care also made it possible for the students to reflect on their actions during and after assignments.

The nursing students reported that the team’s clinical reasoning was conducted verbally from the point at which the EMCC dispatched the assignment, and they rode together in the ambulance. Based on the information sent out by the EMCC, i.e., the patient’s symptoms and level of priority, the nursing and medical students discussed and analysed out loud possible diagnoses, treatments, and symptom alleviation based on their theoretical knowledge and practical experience. The nursing students described that maintaining clinical reasoning, where both students participated by talking and actively listening, created possibilities for knowledge exchange and shared understanding before, during and after the care encounter. The nursing students discussed how they learned from the medical students by listening and asking questions and were able to increase their knowledge in areas such as pathophysiology and pharmacology. They also discussed how medical- and caring science were integrated into a whole through their collaborative clinical reasoning. The nursing students expressed that they contributed with their knowledge while planning care collaboratively with the medical students, who in turn listened and asked questions. The student team created a sense of preparedness for the patient encounter on the scene through their pre-arrival discussions which included division of roles and tasks and possible measures to be taken.

Arriving at the scene, the situation was sometimes completely different from what the students had prepared for. The nursing students discussed how meeting with the unexpected required the team to adapt their plan of action and mindset. To be able to handle the new situation and to learn within it required a sense of openness and safety in the collaboration to maintain the student team’s participation. A sense of safety was achieved through student and supervisor participation in clinical reasoning and reflection before, during and after patient care. Collaborative participation in the team was characterised by both questioning and confirming the plan of care. The nursing students discussed that equality in the learning environment supported a sense of safety and openness since they went out in pairs with no given or expected role in the ambulance service. Without an expected role model to follow, it became possible for the students to use situational leadership, where the one with the knowledge required in the situation became the team leader. The nursing students reported that situational leadership was a new experience for them and initially made them feel insecure. They took the lead when they had more knowledge about the setting and the care required at scene. In doing so, the nursing students felt that they learned and received confirmation of their own capability to be a team leader.

At scene, the nursing students described how collaborative clinical reasoning continued during the patient assessment and decision making. Learning and collaboration occurred when both students explained the causes of symptoms and plan of care for patients and/or next of kin. Some of the learning activities discussed by the nursing students included when the medical students explained how they assessed an electrocardiogram, and when the nursing students gave instructions on how to insert a peripheral venous catheter. After assessing the patient’s symptoms and initiating treatment, the optimal level of care was discussed and decided upon by the team in collaboration with patients and/or their next of kin. When non-conveyance was decided, the team collaboratively gave self-care advice from their respective professional perspectives. In cases where a decision was made to transport the patient to the next level of care, clinical reasoning continued during transit, when the students invited the patient into discussions by providing medical and caring advice and explanations. According to the nursing students, this added a new aspect to the process of learning from each other’s knowledge.

The handover to next level of care, e.g., to the ED, was also done together. Both medical and nursing aspects were highlighted, and the nursing students reflected that this led to a greater understanding of patient needs. After the handover there was an opportunity for reflection on and evaluation of the completed assignment. The nursing students discussed that when these reflections occurred the next assignment became easier to carry out, and it also improved the collaboration. The nursing students also discussed the essential need for continuous reflections and that when one of the students did not want to reflect the IPL failed.

### Factors facilitating learning and collaboration in the ambulance service

The nursing students identified and discussed factors that were vital for facilitating IPL and collaboration. These included the clinical supervisor’s level of skill, the variation of the assignments, and being allowed to act collaboratively with other professionals in the EMS.

The nursing students discussed and agreed that the clinical supervisors were essential for supporting IPL. The students stressed that there was variation between the different clinical supervisors’ motivation, willingness, preparedness, knowledge, and experience in facilitating IPL. The students stated that a good clinical supervisor had the knowledge and ability to step back while on the scene and let the student team step forward. The supervisor facilitated reflection, learning, and deeper knowledge when they let the students assess patients’ need of care together and confirmed or questioned the students’ suggestions on treatment and on transport of the patient to next level of care. The students also discussed that the supervisors facilitated IPL by opening up and challenging the student team to reflect, by asking follow-up questions about their actions during the chain of care. Their questions focused confirming or challenging the what, the how, and the why.

Some of the supervisors encouraged the students to engage in structured reflection on their actions (author remark: Kolb reflective cycle was used) after the assignments. These reflections, according to the nursing students, served to help them understand their own actions, especially when the clinical supervisors highlighted points that the students did not remember or had not noticed during the assignment. The students also gave examples of the opposite, i.e., when the supervisors did not facilitate IPL in the student teams. For instance, some supervisors did not see the point of IPL and when this happened, the nursing students discussed that they were happy to be two students during the shift, in that way they could learn from each other.

The variation in the ambulance assignments was discussed among the nursing students as an important factor in facilitating learning and collaboration. Even if the students could not be the one making the assessment or acting as team leader in violent or life-threatening situations, they learned by observing, reflecting, and being given the opportunity to asking follow-up questions when the assignment was completed. The variation in assignments, along with the fact that no medical records were available and nobody with medical knowledge had assessed the patients physically before entering the scene, presented opportunities for the students to discuss and identify differential diagnoses and diverse methods of symptom alleviation and treatment, and give health care recommendations under guidance by the clinical supervisors. The nursing students discussed that this was something that they had never experienced before during clinical rotations. According to the nursing students’ discussions, the IPL and collaboration was dependent on the medical students’ knowledge, experience, social skills, and their willingness to discuss and learn together. Without these, the learning possibilities decreased and became dependent on the clinical supervisors and/or other actors in the EMS instead.

IPL was facilitated by other actors in the EMS, according to the nursing students. The teams were allowed to act and reflect during the collaboration with the physician-manned units, and in those care encounters all of the various knowledge areas in the team were utilised in order to meet the patient’s needs.

### Medical students’ experiences and self-assessed competencies in interprofessional collaboration

Out of 40 eligible medical students, 72.5% (*n* = 29, female *n* = 16 and male *n* = 13) aged 21–32 (mean 22.4) years answered the questionnaire. When they began their placement in the ambulance service, a total of 22 students (75.9%) had previous experience of IPE, which included participating in simulations, seminars, and/or clinical rotation at IPTWs.

Fifteen of the students (51.7%) answered the open-ended question: *‘Overall, what is your experience of your and the team’s interprofessional collaboration during your clinical rotation in the ambulance service?’*. All commented that they were satisfied with the experience and described that the IPL had given them the possibility to learn and train in interprofessional collaboration. However, they also frequently felt that the clinical rotation had been too short. *‘Very nice and instructive clinical rotation. A weekly placement would be great, 2 days is too little’*. The mean scores on a group level showed that the medical students assessed their competencies in interprofessional collaboration > 3.0 (min1- max 4) in all domains except for ‘Relationship with patients and next of kin’ (2.8) as displayed in Table 2. For additional item level results, see Table 3.

**Table 2:** Domains of interprofessional collaborative competencies

	Group scores mean (range)	Missing
Communication (4 items)	3.3 (3.0–3.5)	–
Collaboration (3 items)	3.3 (3.3–3.4)	–
Team functioning (5 items)	3.2 (3.0–3.5)	–
Role and responsibilities in the IPL (5 items)	3.2 (2.9–3.6)	2
Patient/client-family centred approach (4 items)	2.8 (2.5–3.0)	2
Conflict management/resolution (3 items)	3.1 (2.9–3.2)	6

**Table 3:** Item level results for interprofessional collaborative competencies

Domain	Item mean/median	(range)	Missing
**Communication**			
Communicates with others in a confident, assertive, and respectful manner.	3.5/4	(1–4)	
Communicates opinion and pertinent views on patient care with others.	3.4/3	(1–4)	
Communicates in a logical and structured manner.	3.1/3	(2–4)	
Explains discipline-specific terminology/jargon.	3.0/3	(2–4)	
**Collaboration**			
Establishes collaborative relationships with others.	3.3/3	(2–4)	
Shares information with other providers.	3.3/3	(2–4)	
Integrates information and perspectives from others in planning and providing care.	3.4/3	(2–4)	
**Team functioning**			
Relationship between team functioning and quality of care.	3.4/3	(2–4)	
Recognition of strategies that will improve team functioning.	3.1/3	(1–4)	
Shares leadership and alternates leadership with others.	3.1/3	(2–4)	
Recognition of themselves as part of a team.	3.5/3	(3–4)	
Contributes to interprofessional team discussions.	3.0/3	(2–4)	
**Roles and responsibilities**			
Describes one’s own role and responsibilities to the team/patient/family.	3.1/3	(2–4)	
Professional judgement when assuming tasks or delegating tasks.	3.6/4	(2–4)	
Responsibility for the failure of collaborative goals.	3.0/3	(2–4)	2
Responsibility for individual actions that impact the team.	3.6/4	(2–4)	
Shares evidence-based or best-practice discipline-specific knowledge with others.	2.9/2	(2–4)	
**Collaborative patient/client-family centred approach**			
Seeks input from patient/client and family.	2.5/2	(1–4)	
Integrates patient’s/client’s and family’s circumstances, beliefs, and values in care plans.	2.8/3	(1–4)	1
Shares options and health care information with patients/clients and families.	3.0/3	(2–4)	1
Advocates for patient/client and family as partners in decision-making process.	2.9/3	(1–4)	
**Conflict management/resolution**			
Considers the perspectives and opinions of others.	3.2/3	(2–4)	2
Seeks clarification in a respectful manner when misunderstandings arise.	3.1/3	(2–4)	2
Manages or resolves conflict with others.	2.9/3	(1–4)	2

## Discussion

IPE has the goal of making healthcare professional ready to collaborate by learning and working together in diverse healthcare settings [1]. Our findings and previous studies show, individuals, teams, and organisational factors can both hinder and facilitate IPC [23]. Teams that support and encourage each other, have respectful interplay, and acknowledge diverse input facilitate IPC [23]. In the context of the ambulance service, some of the challenges include team that vary daily, a context that can be unpredictable, and the team being required to make decisions in various situations with limited support [15, 24]. The nursing students’ narratives in this study highlighted that the ambulance service context can offer both positive and negative experiences and that students, colleagues, and supervisors are regarded as central in affecting learning, findings which are similar to those of previous studies [25, 26].

IPE entails a wide range of activities where the teams often consist of nurses and physicians, on both a pre-graduate and continuous level of education and where participation is at the core of learning together [6, 7, 9]. The students’ experiences of learning together in the ambulance service reported in this study indicate that even contexts not previously used for IPE and IPL offer possibilities for interprofessional learning. Overall, our study findings suggest that the context of the ambulance service seemed to facilitate IPL and provided significant opportunities for collaborative learning.

Students’ responses in the current study suggest that the setting presented good opportunities to learn together, since they faced a broad variety of situations and had opportunities to follow patients through the chain of care; the latter was confirmed in other studies [12, 13]. The team got to care for both conveyed and non-conveyed patients, and the students collaboratively reasoned together with patients, family members, and supervisors to reach an understanding. Lederman et al. suggest that making an accurate non-conveyance assessment is like putting a puzzle together with others where the ambulance team must obtain a holistic picture of the patient’s situation [17].

IPL is a joint and interactive process [4] where the learners initiate, analyse, negotiate, and reach conclusions together [11, 27]. The nursing students in the current study felt that maintaining clinical reasoning out loud with the medical students throughout the chain of care was at the core of analysing their different professional knowledge and reaching a common understanding. When meeting with the unexpected, they adapted their mindset, plan, and actions together. Through participating in joint clinical reasoning, they learned together through listening, asking, and questioning. Since the students had little or no prior contextual knowledge, this common starting point in an unfamiliar environment led to an openness between the students, thereby affecting their approach towards the learning situation, as previous discussed by Prosser and Trigwell [28]. The assumption that participation [29, 30] provides a strong incitement for learning is coherent with the fact that the collaborative aspects of learning were described as essential by the nursing students. The two student groups united in their focus on the contextual assignment, i.e., providing care for patients in the ambulance service. Both student groups were unfamiliar with the context and the IPL environment, but their mutual goal of providing care to patients could be regarded as a catalyst for knowledge creation, hence increasing their learning [30]. The content, level, and focus of knowledge creation is largely dependent on both students’ approach to the situation and the context provided, both of which varied to some extent in our results. This knowledge creation is an interpretation of the results that suggests that the students together created possible solutions to their contextual problems as a mutual quest rather than sharing pre-existing knowledge. An assumption is that a large part of the students’ learning was based around the assignment as a quest, rather than the learning of each other’s professions. However, it seems that the students needed to integrate their different areas of knowledge to complete the assignments.

The supervisors are present in care encounters in the ambulance service [12, 13], and in IPL they facilitate learning by gradually stepping back and creating space for the team to take control [11, 27]. The nursing students in this study expressed emphatically that the learning environment was dependent on the role of the supervisor and that the supervisor’s facilitation skills had a great impact on learning. Whilst the clinical supervisor’s contextual knowledge was needed to form the contextual base for IPL, the nursing students reported that when the supervisor intervened less, this increased collaborative learning among students. This supports the assumption that facilitation of IPL is complex [31] and supervisors ensure equal participation, set realistic goals, and offer learners opportunities to reflect [2]. Commonly, clinical supervisors in the ambulance service have little training in facilitating IPL, indicating a need to provide further education and training for supervisors. The medical students’ self-assessment showed that they perceived themselves to be competent in almost all dimensions of their IPC competencies, something that was not always in line with the nursing students’ narratives. Nevertheless, among some students the collaboration was regarded as poor or insufficient, which leads us to believe that when relationships between students were not well established, it does not matter how good self-assessed competence is. Hierarchical structures of power and stereotypes that regard nurses as assistants and physicians as decision makers have been described in previous IPL research as a serious inhibitor of IPL [31–33]. In this study, from the nursing students’ perspective, hierarchical structures of power and stereotypes in the ambulance service were primarily unknown and roles undefined, the learning environment facilitated a sense of safety and openness for developing situational leadership where the student with the knowledge required in the situation became the team leader. This was something new and unexpected for the nursing students, but in this new situation they received confirmation of their own leadership abilities. The medical students assessed themselves as competent in the ability to alternate leadership with others, findings that may confirm that the context facilitated training in situational leadership. It is recognised that the medical perspective is sometimes dominant in the health care organisation [31, 34], and Fewster-Thuente [35] suggests that nursing students need to increase their recommendations on care since they spend more time with patients. In this study, the two groups of students spent equal time with the patients, and it became natural to hand over and give recommendations for further care in collaboration. Both the nursing and the medical students described equal contribution to interprofessional team discussions. This may have contributed to an increased sense of equality between the two groups of students through enhanced collaborative participation in the IPL activities.

### Limitations

The findings from this study should be interpreted in the context of the following limitations. The number of participants could be considered as low, although all eligible students were invited to participate, and the response rate was > 70%. Another limitation was that the selection of participants for group discussions was based on students’ availability, and the medical students were unable to participate due to scheduling issues. Therefore, a questionnaire containing open questions was used as an alternative to gather data on the medical students’ experiences of the IPL. The medical students did not have the possibility to elaborate on their answers through discussions with peers. Despite these limitations, the merging of these findings illustrates both nursing and medical students’ perspectives on IPE in the ambulance service. However, based on the answers to the questionnaire, it cannot be concluded whether the medical students increased their competencies in interprofessional collaboration during their IPL placement. Nor do the students’ answers to the questions provide us with any information about their thoughts on what they learned, or their opinions about the nursing students and their practice.

## Conclusion

The students’ experiences show that the ambulance service offers possibilities for IPE and IPL. Interprofessional learning in the ambulance service was influenced by multiple factors, where the context in conjunction with adequate facilitation by supervisors proved to be essential for IPL. The ambulance service enhanced the students’ learning in an unfamiliar environment, encouraging them to develop collaborative learning strategies and situational leadership regardless of established hierarchical structures and stereotypes that are sometimes present in other parts of the health care service.

## Data Availability

The data analysed during the current study are available from the corresponding author on reasonable request.

## Abbreviations

EMCC: Emergency medical communications centre
EMS: Emergency medical service
EMT: Emergency medical technician
ICAR: Interprofessional Collaborator Assessment Rubric
IPE: Interprofessional education
IPL: Interprofessional learning
IPC: Interprofessional collaboration
IPTW: Interprofessional ward
RN: Registered nurse
